# Perceptions of malaria and acceptance of rapid diagnostic tests and related treatment practises among community members and health care providers in Greater Garissa, North Eastern Province, Kenya

**DOI:** 10.1186/1475-2875-13-502

**Published:** 2014-12-17

**Authors:** Emma Diggle, Ramin Asgary, Georgia Gore-Langton, Erupe Nahashon, James Mungai, Rebecca Harrison, Abdullahi Abagira, Katie Eves, Zoya Grigoryan, David Soti, Elizabeth Juma, Richard Allan

**Affiliations:** The MENTOR Initiative, Crawley, UK; New York University, New York, NY USA; The MENTOR Initiative, Garissa, Kenya; UNICEF, Garissa, Kenya; Department of Malaria Control, Ministry of Public Health, Nairobi, Kenya

**Keywords:** Malaria, RDT, HCW, Community members, Perceptions, Discord, Greater garissa, Kenya

## Abstract

**Background:**

Conventional diagnosis of malaria has relied upon either clinical diagnosis or microscopic examination of peripheral blood smears. These methods, if not carried out exactly, easily result in the over- or under-diagnosis of malaria. The reliability and accuracy of malaria RDTs, even in extremely challenging health care settings, have made them a staple in malaria control programmes. Using the setting of a pilot introduction of malaria RDTs in Greater Garissa, North Eastern Province, Kenya, this study aims to identify and understand perceptions regarding malaria diagnosis, with a particular focus on RDTs, and treatment among community members and health care workers (HCWs).

**Methods:**

The study was conducted in five districts of Garissa County. Focus group discussions (FGD) were performed with community members that were recruited from health facilities (HFs) supported by the MENTOR Initiative. In-depth interviews (IDIs) and FGDs with HCWs were also carried out. Interview transcripts were then coded and analysed for major themes. Two researchers reviewed all codes, first separately and then together, discussed the specific categories, and finally characterized, described, and agreed upon major important themes.

**Results:**

Thirty-four FGDs were carried out with a range of two to eight participants (median of four). Of 157 community members, 103 (65.6%) were women. The majority of participants were illiterate and the highest level of education was secondary school. Some 76% of participants were of Somali ethnicity. Whilst community members and HCWs demonstrated knowledge of aspects of malaria transmission, prevention, diagnosis, and treatment, gaps and misconceptions were identified. Poor adherence to negative RDT results, unfamiliarity and distrust of RDTs, and an inconsistent RDT supply were the main challenges to become apparent in FGDs and IDIs.

**Conclusion:**

Gaps in knowledge or incorrect beliefs exist in Greater Garissa and have the potential to act as barriers to complete and correct malaria case management. Addressing these knowledge gaps requires comprehensive education campaigns and a reliable and constant RDT supply. The results of this study highlight education and supply chain as key factors to be addressed in order to make large scale roll out of RDTs as successful and effective as possible.

## Background

Despite being preventable and treatable, malaria continues to be a life-threatening disease resulting in high levels of morbidity and mortality. Malaria is estimated to cause between 660,000 and over a million deaths every year and in 2012 there were an estimated 207 million cases of malaria [1]. Worldwide estimated malaria mortality rates between 2000 and 2012 fell by 42% across all age groups and by 48% amongst children under five years old [2]. However, the pace of this decrease slowed between 2011 and 2012 [2]. Current day malaria cases and deaths are indicative of a lack of access to indoor residual spraying (IRS) and long-lasting insecticidal nets (LLINs) using effective insecticides, and artemisinin-based combination therapy (ACT) amongst millions of people currently at risk of malaria.

### Malaria diagnosis

Until recently, conventional diagnosis of malaria has relied upon either clinical diagnosis or microscopic examination of peripheral blood smears [3]. These diagnostic methods require trained staff, expensive and fragile equipment, and, in the case of microscopy, an electricity supply. These requirements and the intrinsic potential for human or technical error have been shown to result in patients who do not have clinical malaria being diagnosed as positive and prescribed anti-malarials [3–7]. This over-diagnosis of malaria and the resultant anti-malarial treatment is a waste of resources in often resource-scarce settings, exposes patients to needless anti-malarial therapy, likely contributes to the development of drug resistance, and may compromise patient trust in health care providers as their condition does not improve upon treatment [8, 9]. The problem of misdiagnosis and over (and under) treatment of malaria provided the impetus for the development of a more reliable, field-suitable, and cost effective diagnostic tool [10, 11]. The malaria rapid diagnostic test (RDT) is the current diagnostic tool which best meets these criteria. In 2010 the WHO recommended that all suspected cases of malaria be confirmed with a diagnostic test prior to treatment [12].

Based on the detection of *Plasmodium* antigens in a small drop of the patient’s blood, these tests are easy to use (requiring only minimal training of health workers). As the name suggests, the result is given almost instantly and is easy to interpret. The accuracy of these tests varies between brands and between location and epidemiological setting, however, on the whole the tests have very high levels of sensitivity and specificity and also high negative-predictive values [13, 14]. The reliability and accuracy of RDTs, even in extremely challenging, remote settings where infrastructures may be broken, health care systems weak and education poor, have made them a staple in malaria control programmes and the first choice malaria diagnosis tool, especially when microscopy is not possible, or suitable [2, 12].

Despite the ease of use and interpretation of RDTs, barriers to their correct use and subsequent results-based treatment remain. A study in southeast Nigeria found that health care workers (HCWs) reported perceiving RDTs as a more effective malaria diagnosis tool than both microscopy and clinical diagnosis, however, some providers experienced difficulties in using the kits and ACT was prescribed to 74% of RDT-negative patients [15]. A similar study in primary health facilities (HFs) in Uganda reported high levels of uptake with more than 90% of eligible patients offered an RDT; there was also a 38% point reduction in anti-malarial prescriptions. Of HCWs interviewed it was reported that 92% believed a positive RDT confirmed malaria, whereas only 49% believed that a negative RDT result excluded malaria infection [16]. Given the high negative predictive value of RDTs, the perception of negative test results not being reliable is a misconception and a hindrance to appropriate treatment.

Studies in to the attitudes and beliefs of health care providers and patients surrounding malaria diagnosis, and RDTs in particular, hope to understand why this misconception, and others of its type, persist. Knowledge and understanding of these barriers to the uptake and correct usage of RDTs aid in overcoming them and improving malaria case management. A study in Nigeria found that providers and community members both recognized malaria RDTs as an important step to correct treatment, however, it was also reported that there were concerns as to the reliability of test results with symptoms being deemed more important than test results [17]. A similar study in Uganda looked at the introduction of RDTs in to registered drug shops and also found a mistrust of negative RDT results. Factors reported to reduce the appeal of RDTs included the cost of the test and the extra time required to be tested [18].

### RDTs in Kenya

**Figure 1:**
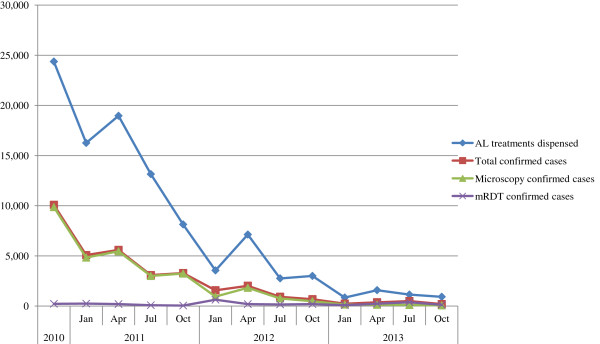
**The total numbers of malaria cases and numbers of artemether lumefantrine treatments distributed.** Total number of AL treatments dispensed, confirmed malaria cases (microscopy and RDT), microscopy confirmed cases, and RDT confirmed cases across the study area between 2010 and 2013 and broken down in to yearly quarters (except 2010 data which was only collected over the period between September and December).

In 2010 the Department of Malaria Control, Ministry of Public Health, Kenya, and the MENTOR Initiative launched the official introduction of RDTs in 42 HFs in Greater Garissa and the districts it contained at the time: Garissa, Lagdera, Fafi, and Balambala. This programme complemented the Ministry of Health programmes supplying ACT to HFs and was intended to both reduce irrational AL consumption and improve prescription practises. The total numbers of confirmed malaria cases and artemether-lumefantrine (AL) treatments distributed between 2010 and 2013 are shown in Figure 1 (Garro C: Evaluation Report; Building Sustainable and Effective Malaria Control for Vulnerable Communitis in Kenya, unpublished); total malaria cases are separated into those diagnosed by RDTs and those diagnosed by microscopy. As the number of RDTs increased in HFs in 2011, there was a marked and rapid decrease in the total number of confirmed cases, the number of microscopy confirmed cases, and the number of AL treatments dispensed. By 2013, the increasing uptake and usage of RDTs in HFs was evident from the number of RDT-diagnosed cases, which was higher than the number of microscopy-diagnosed cases. Reflecting the reductions in total confirmed cases, the total number of AL doses dispensed in 2013 was at a four-year low of 4,519 treatments, down from the peak of 56,511 treatments dispensed in 2011.

### Aim of this study

Knowledge of community members’ and HCWs’ thoughts and opinions on RDTs is vital; it is the aim of this study to understand perceptions towards RDTs and how these affect RDT use, subsequent HCW prescription practises, and patient adherence and behaviour. Results of this kind will be vital in improving the uptake and usage of RDTs in similar contexts in Greater Garissa and other counties of Kenya.

## Methods

### Study area and population

Garissa is home to an estimated 623,060 people, over 90% of which are ethnic Somali Kenyans, many of whom are nomadic pastoralists, living in an area of 44,175 sq km [19]. According to the last demographic health survey, the literacy rates in North Eastern Province are the lowest in Kenya at 21% for women and 64% for men [20]. The county has, for the last 20 years, hosted the world’s largest refugee camp, which by 2013 contained an estimated 596,000 refugees, largely from neighbouring Somalia [21].

**Figure 2:**
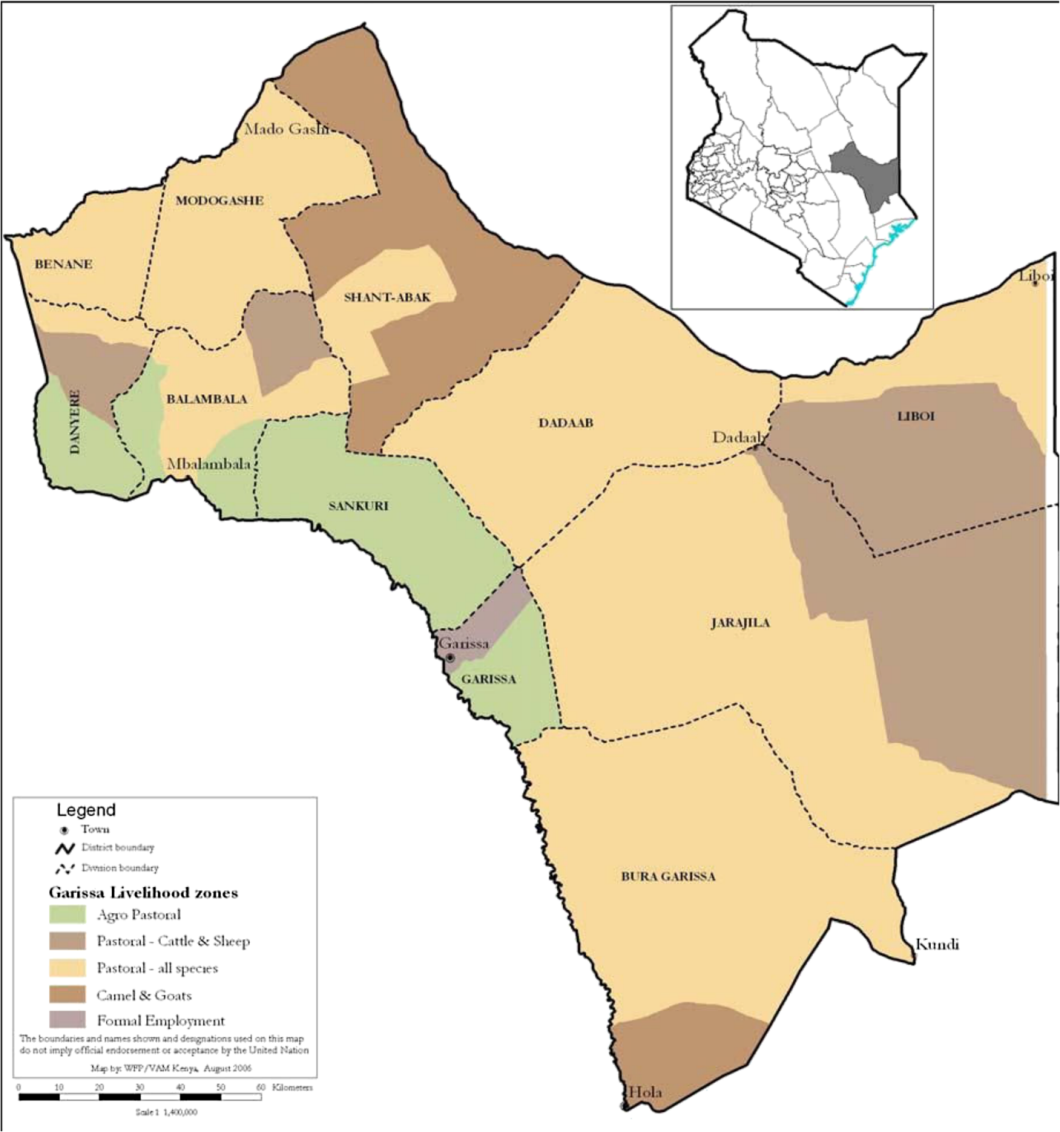
**Map of the study area.** A map of the study area of Greater Garissa, North Eastern Province, Kenya [30].

The study was conducted in the four original districts of Garissa County: Garissa, Balambala, Lagdera, and Fafi, to which Dadaab was subsequently added (Figure 2). The area suffers regular rebel attacks and is generally very insecure [22, 23]. The study area is prone to malaria epidemics [24]. Immunity is low due to infrequent exposure to infection during the early years of childhood and the occurrence of seasonal, severe flooding resulting in sudden increases in mosquito populations. In areas such as these with typically seasonal epidemics the community is often highly aware of the threat of malaria and in many cases both communities and health workers have come to associate all fevers with malaria despite the fact that outside of the rainy season malaria is a very minor cause of febrile illness [25]. Many other diseases (bacterial, viral, parasitic) which present with fever commonly occur in the same area [26–29].

In this area, the MENTOR Initiative provides malaria services in partnership with the Ministry of Public Health. The study was performed by the MENTOR Initiative as part of a quality assurance assessment and the continual process of monitoring and evaluation.

### Study design

HFs (all outpatient facilities) were selected using purposive and criteria sampling technique as well as probability sampling. Due to chronic security issues and lack of accessibility during the study periods, five of the HFs were not included; a total of 12 HFs were sampled from a maximum of 42 facilities in the area. Six were chosen on the basis of whether or not they were accessible, and their rates of over-prescribing ACT (defined as more than 2.5%) *versus* six with good prescription habits, defined as ACT over-prescription rates below 2.5% (according to data collected by the MENTOR Initiative from May 2011 to July 2013). However, due to security issues the health facilities also had to be sampled based on accessibility. HFs in an area deemed to be insecure or inaccessible were not visited.

Prior to data collection, five pilot evaluations and discussions with local key informants were used to highlight key issues and to make sure they were covered in study interviews. These interviews were used to help probe questions and areas of focus, and to ensure the cultural adaptability, reliability, and validity of the study design and questions. The interview tools were informed based on a previous study in the Mandera region of Kenya [31] and subsequent pilot evaluations performed during ongoing implementation of the MENTOR services/programme over several months prior to the study implementation. This pilot evaluation included informal discussions with local key informants and was used to highlight key issues to be covered in study interviews.

FGDs and interviews were held at HFs, held separately for each group of participants and were conducted mostly in Somali (the most commonly used language) with some in Swahili, and using trained interpreters or English when applicable. FGDs and IDIs were tape-recorded and written notes were taken on non-verbal communication during interviews. Active discussion and interaction among participants was encouraged. Participants were asked a series of open-ended questions with subsequent probes and follow-up questions as needed. The decision to choose FGDs over individual interviews for community members was based on input from community leaders and local informants. A mixture of male and female interviewers originally trained as either clinical officers, CHWs or pharmacist and trained in how to conduct the interviews for this study by ED conducted the interviews over a period of 17 days. The sex of the interviewer was always the same as the sex of the interviewee.

The selection criteria of participants varied depending on the data collection method and the type of participants. Key informants were defined as persons positioned to possess knowledge of particular relevance to the research themes, including community health workers (CHWs), medical providers, religious figures, and community leaders who were working or living in the catchment area for this study. Community member participants were selected from patients aged 18 years or older, with a febrile illness, who presented to HFs on the date of the interview. Fever status was defined if recorded in the medical record (either reported by patient or measured by HCW). Both male and female subjects were included. HCWs (n = 65) from the same HF were invited to participate and key informants were selected using criteria sampling technique including different occupations, religious backgrounds, age groups, and gender.

FGDs were performed with community members recruited from healthcare centres supported by the MENTOR Initiative, as were in-depth interviews (IDIs). FGDs with patients explored their understanding of malaria causes, transmission, signs and symptoms, diagnosis, diagnostic tools, and treatment. IDIs with HCWs explored their perspectives and experiences of using malaria diagnostic tools, including RDTs, prescription and treatment practises, and experience with patients. Additionally, interviews with key informants were conducted.

### Ethical approval

Oral consent was obtained, and participants were informed that their participation would have no impact on their treatment or affect them otherwise, and that they could retract their responses and/or participation at any time. Ethical approval was obtained from the Kenyatta National Hospital and the University Of Nairobi College Of Health Sciences (P624/11/2012).

### Analysis

Interview transcripts were coded and analysed for major themes. Coding of transcripts was done using Excel and through open, axial and selective coding. More than 600 pages of transcripts were analysed and around 190 codes were developed. Codes fell into distinct but overarching categories. Patterns in responses and codes were analysed to explore and develop relevant themes. Observational data, including setting and non-verbal communication, were collected and noted in order to enhance analysis. Inductive grounded theory process for analysis was applied. During content analysis preliminary coding was developed based on priority codes derived from the theoretical framework and conceptual model guiding the study. Critical deliberation was performed about initial coding and reviewed coding for similarities and variations among coders’ output. Discrepancies were discussed and a high level of agreement was achieved, suggesting that the coding scheme used was appropriate. Two researchers independently reviewed all codes, then met and reviewed codes, discussed the specific categories, used inputs as needed, and finally characterised, described, and agreed upon major important themes.

## Results

### Demographics of study population

A total of thirty-five FGDs were carried out with a range of two to eight participants (median of four), thirty-four of these FGDs were with patients, and one was with HCWs (Table 1). Of the 157 community members, 103 (65.6%) were women. The youngest participant was 18 years old and eight participants were above the age of 50. The majority of participants were illiterate and the highest level of education was secondary school. Some 76% of participants were ethnic Somali. Of the female community participants the overwhelming majority were reported to be housewives. The full demographics of key informants and HCWs are presented in Table 2.

**Table 1:** **Data collection methods and participants involved**

Data collection method (n)	Participants (n)
FGD (1)	HCWs (8)
FGD (34)	Patients (157)
IDI (23)	HCWs (23)
KI interviews (27)	CHWs, medical providers, religious figures, community leaders (27)

Summary information of the data collection methods used and the numbers and types of participants involved.

**Table 2:** **Demographics of health care workers and key community informants**

Health care workers	No.	% (Total = 23)	Key informants	No.	% (Total = 27)
**Profession**					
Clinical officer	4	17.4	Cultural Leaders (Imam and Village Elder)	3	11.1
Nurse	9	39.1	Group Leaders (youth, women’s group, community information officer, prison leader, school teacher, college student)	10	37.0
Laboratory technician	9	39.1	Administrative Health Key Informants (Chairman Health Centre, Hospital Committee Member)	3	11.1
Pharmacist	1	4.3	Other health worker (community health workers)	6	22.2
			Service industry (business man, tea vendor, private pharmacy owner, chairman water project)	5	18.5
**Age**					
30 and under	15	65.2	30 and under	13	48.1
31-41	8	34.8	31-41	10	37.0
42-51	0	0	42-51	3	11.1
**Ethnicity/tribe**					
Somali	1	4.3	Somali	20	74.0
Other	20	87.0	Other	6	22.2
Unknown	2	8.7	Unknown	1	3.7
**Gender**					
Female	8	34.8	Female	7	25.9
Male	15	65.2	Male	20	74.0
**Years of professional training**			**Level of education**		
3 years and above	23	100	Illiterate	4	14.8
Fewer than 3 years	0	0	Primary School dropout	7	25.9
			Primary School complete	3	11.1
			Secondary School dropout	7	25.9
			Secondary School Leaver	4	14.8
			Diploma	1	3.7
			Unknown	1	3.7

Demographic information, including the profession, age, ethnicity/tribe, gender, and years of professional training, separated for health care workers and key informants.

### Community members

Overarching themes surfaced from FGDs relating to general knowledge about transmission, symptoms, treatment, expectations of diagnostic and treatment methods, treatment adherence, and medication taking practices.

### General knowledge about malaria and transmission

Community member participants had varying levels of understanding about the causes of malaria. The majority of participants understood mosquitoes to be at least one cause of malaria, but most did not correctly identify the pathway of transmission. Other attributed causes included water, food (mangoes in particular), milk, dirt, poor hygiene, and exposure to someone who has malaria (via breathing/sharing rooms).

“*Drinking dirty water causes malaria*” (FGD B1, R4).

“[Children] *will get through their breath if they sleep together*” (FGD M3 R3).

Some participants believed malaria could be caused by various other illnesses, such as a cold that has worsened, or generalized “excess bile”.

“*Malaria is caused by being sick severely and not visiting health facility”* (FGD B1, R2*)*

*“We just know bile to be the cause of malaria not as in hospitals…. describe it”* (FGD B3 R2).

Alternatively, malaria itself was said to cause other illnesses.

“*Malaria is the starting point of all disease…after that everything else changes to other diseases*” (FGD B3 R1)

Among participants who connected mosquitoes with malaria, some discussed the use of nets, and neglecting to use them properly as a way to get malaria. Generally, participants mentioned more than one cause, and there was a wide range of responses. Nets were discussed as a means of prevention, as were other methods, such as showering after a mosquito bite.

“*Take shower early in the morning after mosquito bite at night to prevent malaria….the blood is not circulated at night and the mosquito bites at night……the malaria is not transferred into the blood yet*” (FGD B4 R3).

### Malaria symptoms and treatment

Regarding malaria symptoms, responses were quite general, although some participants did state that malaria could be differentiated from other fevers and illnesses.

*“The strongest fever it’s for malaria*” (FGD M6 R4)

“*I know myself if I have malaria, I will feel dizziness*” (FGD GKP5 R3)

Symptoms commonly identified included fever, chills, body aches, headaches, joint pain, weakness, appetite loss, fatigue, and a cough. Most participants acknowledged that fever and many of these symptoms may come from other diseases (typhoid, whooping cough, tuberculosis, pneumonia, etc.).

Seeking treatment right away *versus* after a few to several days of alternative therapy depended on how close or accessible the hospital or health centre was. Participants who lived closer to the hospital responded that

“*immediately I become sick that is when I come to hospital”* (FGD M6 R8).

While others noted that they go to the treatment facility

“*only when I have the money and time to visit”* (FGD Me4 R2).

“*People who live in rural areas… don’t visit the facility right away when they’re sick but after 7 days… some become critical… then they are brought the facility by donkey cart or camel… [or] car*” (FGD B2 R1).

### Expectations of diagnostic and treatment methods

Almost all participants were familiar with microscopy and RDT, although many had not actually seen these tools used, and some noted scepticism.

“*I don’t believe that one… microscope”* (FGD M3 R3).

“*I will not believe that [negative diagnosis if child presents with symptoms]”* (FGD M3 R1).

A few participants noted a preference of one method over the other. However, when discussing malaria diagnosis, more participants said they wanted malaria treatment, even in the case of a negative diagnosis by either RDT or microscopy.

“*I will like him to give me ACT [when testing negative for malaria]*” (FGD B1 R4).

“*But I still have malaria….am having loss of appetite*” (FGD B1 R2).

“*Sometimes some of us insist that the doctor treats us for the same malaria that was negative*” (FGD If6 R1).

A common response when asked why they still wanted the treatment in light of a negative diagnosis was that malaria medication

“*will cure the joint pain*” (FGD B1 R4).

Some participants acknowledged that this was a pervasive attitude in the community, and pointed out that diagnosis is important and should be trusted. Some also talked about how attitudes towards testing are changing, and that RDT is becoming more accepted and encouraged. Nonetheless, its resemblance to HIV testing kits poses a problem, as some patients fear they are being tested for HIV, or they believe it is not the right test.

“*I prefer microscopy because RDT I don’t know whether they are testing HIV or something else*” (FGD GKP4 R1).

Despite some changing attitudes, there is still resistance in the community to accepting negative diagnosis, with patients diagnosed as negative getting malaria medication either from friends/relatives or from private pharmacy shops.

“*If he doesn’t give me, I will just buy*” (FGD GKP5 R3).

Participants recounted that it is common to expect a positive diagnosis, and to not accept or trust a negative RDT result. Local pharmacy shops make it easy to obtain malaria treatment, and make it available by request (without use of RDT or microscopy).

“*If [test at hospital] tells you are negative maybe there is possibility of the instrument he is using might be faulty*” (FGD If4 R1).

Participants were aware of the different types of treatment available for malaria. Alternative therapy was also discussed. These included the use of neem tree leaves, camel’s milk, aloe vera, and other methods. Attitudes towards medication adherence, once malaria treatment is prescribed, were inconsistent. A common response was stopping treatment once one would feel better

“*I stop [the medication] when he [the child] is fine*” (FGD B5 R2)

in order to save the rest of the medication for another time, or to share with a relative or friend if they think they have malaria.

“*I ask her of the sign and symptoms and they are same as mine then I will share it with her*” (FGD GKP5 R2).

Another reason for non-adherence was simply that taking the medication was unpleasant or too cumbersome when taking too many pills at a time. Several respondents pointed out that they wished for extra medication due to the difficulties of getting to the health centre in the future.

“*If you are not living near dispensary you would wish the doctor to give you excess so that you give your children since you are not be able to come another time”* (FGD M3 R3).

### Health care workers

#### General perceptions of RDTs and microscopy

General themes circled around advantages and disadvantage of RDTs and microscopy, patient pressure and treatment expectations despite negative test results, issues of adherence, lack of consistent supplies, and the importance of patient health education. HCWs generally found both microscopy and RDT as effective tools for malaria diagnosis, each with their own advantages and disadvantages. The RDT was noted for its ease and swiftness of use, portability and non-reliance on electricity. Microscopy, for its familiarity, ability to provide more information about the infection and opportunity to discern the organisms present or identify other illnesses.

“[*With microscopy] you can really see with your eyes that this is a real parasite if you are somebody really trained*” (DHMT R4).

However, microscopy requires a higher level of training, and is subject to the skill of the technician more than RDTs.

“*All health workers can [use] RDTs… it’s a procedure that anybody can perform. Unlike the microscopy whereby we only have the lab technician to perform it. RDT is simple*” (IDI R7).

Notably, when discussing the different diagnostic tools, health workers consistently pointed out that use of tools is subject more to availability and access than provider preference. They elaborated on inconsistent supply, resulting in RDTs not always being available for testing. They also noted a level of scepticism towards RDTs, both among patients and clinic staff, due to their surprising high rate of negative results, as compared with what both patients and providers were used to seeing when only symptoms were used for diagnosis.

“*If the RDT turns negative and you still see this patient has those signs of malaria is better that you send that patient for microscopy for further testing*” (IDI R9).

#### Provider-patient interactions

With regard to patient reactions to and expectations of diagnosis, two major themes emerged. It was widely reported that patients do have expectations of being treated for malaria regardless of diagnostic result.

“*Whenever you test and you find malaria is negative, you find that person is not satisfied*” (IDI R2).

Most HCWs discussed how this is highly prevalent and problematic in their community, and how providers in the past have overprescribed malaria treatment in part due to these expectations.

“*They believe that every fever is malaria regardless*… *Even us whenever we used to have fever we used to think it is malaria but when we do RDT, it is negative*” (IDI R1).

At the same time, most noted the role of the provider to educate the patient, and reported good outcomes from taking the time to speak with patients and explain to them how malaria is transmitted, how testing works and the possibility of other illnesses causing their symptoms. They reported positive experiences when they tried

“*to make [patients] understand that not all the fever… to be malaria. We counsel before the test, and tell them, let us [perform] test…but it can be negative, it can be positive. So if it is negative it doesn’t mean you don’t have any problem… but we have to rule out malaria first…and they appreciate, they really appreciate*” (IDI R2).

HCWs discussed their impressions of community perspectives. Many patients they see think that fever automatically means malaria, if not all year, then in certain seasons when malaria is perceived to be more prevalent. They discussed community misconceptions about transmission, such as food sources and drinking dirty water. The community does not trust RDTs enough, particularly when they see a negative diagnosis.

“*When you test… they will start even laughing and they will say I know it will still come negative so there is no need for you to test*” (IDI R3).

Health workers reported that patients often either insist on being given malaria medication, or they go to their local shops where they can purchase malaria medication anyway (without diagnosis). They also discussed problems with adherence, and practices such as splitting up medication with relatives or friends, or saving half of their treatment course for another time.

#### Suggestions for improvement

Moving forward, health workers emphasized that patients and the community at large need to be educated about malaria, its transmission, diagnosis, and treatment, as well as about other febrile illnesses.

“*I think again there is need for mobilization so that we can provide health education to them so that they can know what really causes malaria not just, oh, the child has fever*” (IDI R18).

Additionally, they discussed the issue of resources. While education and health promotion is important in facilitating community attitudes, they also pointed out that supplies of both diagnostic tools and treatment/medication must be consistent.

“*You know like right now we have … some days we run out [of supplies]”* (IDI R3).

Health providers also discussed continued training needs, particularly in diagnostic methods such as microscopy.

## Discussion

The study and results presented here explore the thoughts and opinions of both community members and HCWs in relation to RDTs in Greater Garissa, which have only been available relatively recently. It is of particular interest and importance to investigate perceptions towards RDTs in this area, and over the initial period of national scale-up. As part of the nationwide rollout of RDTs in Kenya, the President’s Malaria Initiative and the Global Fund procured a total of 8.6 million RDTs in September 2012 for use in dispensaries and HFs. At the baseline measurement in 2010 only 8% of HFs had RDTs, compared to the 31% stocking the tests in 2012 [32]. In order to make this continuing national scale-up as effective as possible in improving malaria diagnosis and treatment, studies such as this one are vital in understanding context-specific challenges that may compromise uptake and usage of RDTs.

The main findings of this study were that there are a number of misconceptions regarding malaria transmission, diagnosis, prevention, and treatment. Participants reported that malaria can be transmitted via food or drink products and via the air. There is an obvious gap in the knowledge of the community with regard to mosquitoes as the vector of transmission. This lack of information or understanding is also present when discussing malaria prevention methods; one participant reported that a morning shower could prevent malaria infection. On the other hand, it is encouraging that there is a general knowledge of the non-specific symptoms of malaria as well as an appreciation of the ability of other organisms to bring about these symptoms. A basic knowledge by community members of transmission and prevention of malaria is integral to ensuring an appreciation of the need for confirmatory diagnosis before the prescription of anti-malarials, and, in turn, a respect for the result of the RDT test and the following anti-malarial prescription or referral.

Whilst it has been shown that the accuracy of RDTs is comparable to that of expert microscopy [33, 34], unless CHWs appreciate the ease and reliability of RDTs, uptake and correct usage may remain low, and over-prescription rates may remain similar to those seen when using microscopy [5, 9, 35–37]. It is thus promising that CHWs expressed an appreciation of the simplicity, rapidness and portability of RDTs over microscopy as well as acknowledging the large bonus of not being dependant on an electricity supply. Whilst all HFs in this study had the equipment and facilities required to carry out diagnosis by microscopy the availability of this service would depend on whether or not the laboratory technician was available. The negative comments pertaining to RDTs related to the perceived advantages of microscopy and the familiarity CHWs feel towards it and the added benefits of more information about parasitic load. The issue of familiarity is one which time and continued RDT use and support and training will address. As for the issue of not being able to measure parasitic loads, this should not be a barrier to RDT use with patients presenting at health facilities. Passive case detection such as carried out in this study relies on patients feeling ill enough to go to a HF and seek diagnosis and treatment. This usually means that the patient has a parasite load high enough so that RDTs can be relied upon to pick up infection. This may not always be the case when actively looking for malaria infections, some of which may have very low parasite densities. All RDT-positive patients require treatment and the measurement of parasitic load is irrelevant in these cases. The message of RDT positive being the principle guide to treatment must be relayed in a clear manner to HCWs and patients.

The issue of acceptability of negative RDTs is one of high importance amongst both community members and HCWs. There has been a long history in Kenya, as in much of Africa, of prescribing anti-malarials on the assumption that most headaches, joint pains and fevers are due to malaria infection [38–40]. The universal test and treat strategy of today is a significant change from previous WHO guidelines recommending presumptive treatment of all suspected cases of malaria; with this recommendation in place until recently it is unsurprising that the prescription of anti-malarials in the absence of a confirmatory diagnosis has long been the norm and continues to be in many areas and HFs [40]. However, as malaria prevalence continues to decrease, the proportion of fevers attributable to a cause other than malaria increases; diagnosis based solely on clinical symptoms alone is not efficient, cost-effective or ethical [11, 38]. In the case of this study, community members report not believing RDTs if they are negative, as they perceive their symptoms alone as confirmation of malaria. This highlights a key shortcoming of RDTs and their ability to only exclude or confirm the presence of malaria parasites, leaving the patient with no more information as to what may be causing their symptoms. Upon a negative RDT result there is great dichotomy between patients who want anti-malarials to alleviate their joint aches, as they know they do from previous experience, and clinicians who want to determine the real cause of symptoms but are not able to do so without other diagnostic tests. The vertical nature of RDTs and the lack of availability of other diagnostic tests in areas where malaria prevalence is low and many other infectious diseases co-exist, acts as a barrier to uptake and acceptability of RDTs as patients and clinicians alike are unable to treat the true cause of the symptoms.

Reporting from both CHWs and patients of suspicion towards the reliability of negative RDTs highlights the need for some form of reassurance for HCW and patient alike. Novel tools such as Positive Control Wells allow for rapid evaluation of RDT performance without the need for cross-checking against expert microscopy, potentially to increasing the confidence of clinicians in the quality of RDTs and encouraging confident management of symptoms according to RDT result [41]. Whilst additional tests, such as these, may provide solid proof of the validity of RDT results they may also just present another tool around which suspicion builds. Probably more suitable and logistically feasible in the setting of this study are the ongoing efforts to educate and encourage CHWs in their trust of RDT results and training in passing this information on to the patient. HCWs who are competent in the use of RDTs and trust the result given should prepare patients for negative results and reassure them that they do not mean the patient will go untreated for the true cause of their symptoms. In areas such as the study setting of Garissa County where transmission is highly seasonal and malaria incidence low over most of the year, this emphasis is of particular importance as the proportion of negative RDTs would be expected to be high.

A highly important point raised by CHWs and related to all the issues discussed thus far is the problem of availability and supply of RDTs. CHWs reported RDTs not being consistently available in the quantities needed. If CHWs are not able to consistently offer RDTs to patients the task of familiarizing patients with RDTs and educating them as to their importance becomes infinitely harder, despite the best efforts of CHWs. Thus the issue of availability is tightly linked to the issue of education and, in turn, RDT uptake and correct usage. It is of concern that this issue was raised by CHWs in this relatively small study area of Greater Garissa; whilst the frequency of stock-outs was greatly reduced during this study period they evidently still occurred often enough to be reported by CHWs. If supply chain issues already exist within this study area of just one county, they are likely to be an even greater challenge when working towards national scale up of RDTs across the whole country. The availability of sufficient supplies of ACT is highly pertinent to this point as the use of RDTs is only logical should ACT then be available for prescription to positive cases. In many public and private HFs in Kenya the availability of affordable ACT cannot be relied upon and has been shown to have been a significant factor in influencing patterns of anti-malarial use [42–45]. Securing and backing up the supply chains of both RDTs and ACT should be a priority in any national programme in order to sustain the trust and proper use of RDTs and ACT amongst CHWs and patients alike.

### Limitations

With the aim of extrapolating study results to other counties of Kenya and informing the national roll-out of RDTs, the main limitation to this study arises due to the study area being just one county. Whilst the HFs, HCWs and community members involved in this study are unlikely to be drastically different from their counterparts in other counties, appreciation of potential differences, and thus potential limitations to the generalizability of the data, is important. It is not unfeasible that the political history and current day security setting of Greater Garissa have had an impact on both the general capacity and functioning of HFs in the county, the health-seeking behaviours of patients, and the quality and consistency of health care provided by HCWs. Any or all of these could have an impact on RDT uptake and correct usage in ways that may not be seen in other counties. Changes in the epidemiology of malaria between, and within, counties are likely to pose another limitation to the use of these results. For example, in the southwest of Kenya, surrounding Lake Victoria, where malaria endemicity is ranked greater than 40%, the proportion of negative RDTs will be much smaller than seen in Greater Garissa. This may well change the knowledge and perceptions of HCWs and patients towards malaria and RDTs. Limitations may also arise due to the qualitative research methods used; whilst study participants were not told the research question or the purpose of the study, it is plausible that their answers and opinions may have been affected by one of many types of bias.

## Conclusions

Misconceptions and incomplete knowledge about malaria transmission and prevention contribute to the under-utilization and misuse of available RDTs. The diagnosis of malaria has changed so drastically in Garissa over the last 50 years; from all fevers being presumed to be malaria to the current requirement of confirmatory diagnosis, that it is unsurprising that despite some understanding of the transmission, prevention, diagnosis and treatment of malaria, gaps in knowledge and incorrect beliefs still exist and may act as barriers to accurate malaria diagnosis and correct case management. Addressing these barriers to uptake and correct usage requires comprehensive and ongoing education campaigns for both providers and communities, which in turn necessitate a reliable and consistent RDT supply. The results of this study highlight education and the supply chain as key factors to be addressed in order to make national scale-up of RDTs as successful as possible. Emphasis should also be placed on the need for diagnostic tests for other causes of illness amongst patients in order to strengthen the entire health system and not just malaria case management. Based on the findings of this study it is recommended that NGOs and national malaria programmes are used to reinforce education for communities regarding malaria misconceptions, improving access to health care at more peripheral levels, education and ongoing refresher programmes for CHWs, and private and public partnership to improve access to diagnostic methods and treatment. Along with these local interventions, it is also recommended that transnational approaches to secure a consistent and reliable supply of RDTs is made a priority. This study highlights the many factors which must be considered when striving to achieve both correct case management of accurately diagnosed malaria and a well functioning health care system which patients can rely on, whether they have malaria or not.

## Abbreviations

ACT: Artemisinin-based combination therapy
AL: Artemether-lumefantrine
CHW: Community health worker
FGD: Focus group discussion
HF: Health facility
IDI: In-depth interviews
IRS: Indoor residual spraying
LLIN: Long-lasting insecticidal net
NGO: Non-governmental organization
RDT: Rapid diagnostic test
WHO: World Health Organization.

## References

[1] Johns Hopkins Malaria Research Institute (2014). About Malaria.

[2] WHO (2013). World Malaria Report.

[3] Wongsrichanalai C, Barcus MJ, Muth S, Sutamihardja A, Wernsdorfer WH (2007). A review of malaria diagnostic tools: microscopy and rapid diagnostic test (RDT). Am J Trop Med Hyg.

[4] Harchut K, Standley C, Dobson A, Klaassen B, Rambaud-Althaus C, Althaus F, Nowak K (2013). Over-diagnosis of malaria by microscopy in the Kilombero Valley, Southern Tanzania: an evaluation of the utility and cost-effectiveness of rapid diagnostic tests. Malar J.

[5] Reyburn H, Mbakilwa H, Mwangi R, Mwerinde O, Olomi R, Drakeley C, Whitty CJM (2007). Rapid diagnostic tests compared with malaria microscopy for guiding outpatient treatment of febrile illness in Tanzania: randomised trial. BMJ.

[6] Mwanziva C, Shekalaghe S, Ndaro A, Mengerink B, Megiroo S, Mosha F, Sauerwein R, Drakeley C, Gosling R, Bousema T (2008). Overuse of artemisinin-combination therapy in Mto wa Mbu (river of mosquitoes), an area misinterpreted as high endemic for malaria. Malar J.

[7] Reyburn H, Mbatia R, Drakeley C, Carneiro I, Mwakasungula E, Mwerinde O, Saganda K, Shao J, Kitua A, Olomi R, Greenwood BM, Whitty CJM (2004). Overdiagnosis of malaria in patients with severe febrile illness in Tanzania: a prospective study. BMJ.

[8] White NJ (2004). Antimalarial drug resistance. J Clin Invest.

[9] Chuma J, Okungu V, Molyneux C (2010). Barriers to prompt and effective malaria treatment among the poorest population in Kenya. Malar J.

[10] Moody A (2002). Rapid diagnostic tests for malaria parasites. Clin Microbiol Rev.

[11] Shillcutt S, Morel C, Goodman C, Coleman P, Bell D, Whitty CJ, Mills A (2008). Cost-effectiveness of malaria diagnostic methods in sub-Saharan Africa in an era of combination therapy. Bull World Health Organ.

[12] WHO (2011). Universal access to malaria diagnostic testing – An operational manual.

[13] WHO (2012). Malaria rapid diagnostic test performance. Results of WHO product testing of malaria RDTs: Round 4.

[14] Selimuzzaman SM, Islam SJ, Nahar Z, Das R, Rahman MA (2010). Malarigen malaria Pf/Pv antigen rapid test: a simple and effective tool for diagnosis of malaria in the far-flung hilly areas of Bangladesh. Mymensingh Med J.

[15] Uzochukwu BSC, Onwujekwe E, Ezuma NN, Ezeoke OP, Ajuba MO, Sibeudu FT (2011). Improving rational treatment of malaria: perceptions and influence of RDTs on prescribing behaviour of health workers in southeast Nigeria. PLoS One.

[16] Kyabayinze DJ, Asiimwe C, Nakanjako D, Nabakooza J, Counihan H, Tibenderana JK (2010). Use of RDTs to improve malaria diagnosis and fever case management at primary health care facilities in Uganda. Malar J.

[17] Ezeoke OP, Ezumah NN, Chandler CC, Mangham-Jefferies LJ, Onwujekwe OE, Wiseman V, Uzochukwu BS (2012). Exploring health providers’ and community perceptions and experiences with malaria tests in South-East Nigeria: a critical step towards appropriate treatment. Malar J.

[18] Chandler CIR, Hall-Clifford R, Asaph T, Pascal M, Clarke S, Mbonye AK (2011). Introducing malaria rapid diagnostic tests at registered drug shops in Uganda: limitations of diagnostic testing in the reality of diagnosis. Soc Sci Med.

[19] **HOME - Garissa County Government** [http://www.garissa.go.ke/]

[20] Kenya National Bureau of Statistics (KNBS) and ICF Macro (2010). Kenya Demographic and Health Survey 2008–09.

[21] **ECHO Factsheet Kenya – June 2013 - Kenya ReliefWeb** [http://reliefweb.int/report/kenya/echo-factsheet-kenya-–-june-2013]

[22] **Timeline: Main attacks since 1998** [http://www.aljazeera.com/news/africa/2013/09/201392294643836478.html]

[23] **Timeline: Attacks in Kenya since offensive against al-Shabaab, 2011–2013 Sabahionline.com** [http://sabahionline.com/en_GB/issues/timeline_kenya_attacks]

[24] Kenya National Bureau of Statistics (2013). Kenya - Kenya Malaria Indicator Survey 2010 - Overview.

[25] Graz B, Willcox M, Szeless T, Rougemont A (2011). “Test and treat” or presumptive treatment for malaria in high transmission situations? A reflection on the latest WHO guidelines. Malar J.

[26] National Travel Health and Network Centre: **NaTHNaC Kenya: Country Information.** [https://www.nathnac.org/ds/c_pages/country_page_ke.htm]

[27] World Health Organization; Global Task Force on Cholera Control (2010). Cholera Country Profile: Kenya.

[28] World Health Organization; Regional Office for Africa (2007). Rift Valley Fever Outbreak.

[29] Berkley JA, Lowe BS, Mwangi I, Williams T, Bauni E, Mwarumba S, Ngetsa C, Slack MPE, Njenga S, Hart CA, Maitland K, English M, Marsh K, Scott JAG (2005). Bacteremia among Children Admitted to a Rural Hospital in Kenya — NEJM. New Engl J Med.

[30] **World Food Programme; Map of Greater Garissa, North Eastern Province, Kenya** 2006.

[31] Asgary R, Grigoryan Z, Naderi R, Allan R (2012). Lack of patient risk counselling and a broader provider training affect malaria control in remote Somalia Kenya border: Qualitative assessment. Glob Public Health.

[32] *President’s Malaria Initiative; Kenya Malaria Operational Plan FY 2014*. Geneva, Switzerland: Foundation for Innovative New Diagnostics; 2014.

[33] Marx A, Pewsner D, Egger M, Nüesch R, Bucher HC, Genton B, Hatz C, Jüni P (2005). Meta-analysis: accuracy of rapid tests for malaria in travelers returning from endemic areas. Ann Intern Med.

[34] Ochola LB, Vounatsou P, Smith T, Mabaso MLH, Newton CRJC (2006). The reliability of diagnostic techniques in the diagnosis and management of malaria in the absence of a gold standard. Lancet Infect Dis.

[35] **FIND - Malaria rapid diagnostic tests: An implementation guide - essentials for RDT implementation** [http://www.finddiagnostics.org/resource-centre/reports_brochures/malaria_rdts_implementation_guide_june2013]

[36] Bastiaens GJH, Bousema T, Leslie T (2014). Scale-up of malaria rapid diagnostic tests and artemisinin-based combination therapy: challenges and perspectives in sub-Saharan Africa. PLoS Med.

[37] Skarbinski J, Ouma PO, Causer LM, Kariuki SK, Barnwell JW, Alaii JA, de Oliveira AM, Zurovac D, Larson BA, Snow RW, Rowe AK, Laserson KF, Akhwale WS, Slutsker L, Hamel MJ (2009). Effect of Malaria Rapid Diagnostic Tests on the Management of Uncomplicated Malaria with Artemether-Lumefantrine in Kenya: A Cluster Randomized Trial. Am J Trop Med Hyg.

[38] Tangpukdee N, Duangdee C, Wilairatana P, Krudsood S (2009). Malaria Diagnosis: A Brief Review. Korean J Parasitol.

[39] Bassett MT, Taylor P, Bvirakare J, Chiteka F, Govere E (1991). Clinical diagnosis of malaria: can we improve?. J Trop Med Hyg.

[40] WHO (2006). Guidelines for the treatment of malaria.

[41] World Health Organization Regional Office for the Western Pacific Region: **RDT Evaluation Programme, Positive Control Wells.** [http://www.wpro.who.int/malaria/sites/rdt/who_rdt_evaluation/control_wells.html]

[42] Watsierah CA, Ouma C (2014). Access to artemisinin-based combination therapy (ACT) and quinine in malaria holoendemic regions of western Kenya. Malar J.

[43] Kangwana BB, Njogu J, Wasunna B, Kedenge SV, Memusi DN, Goodman CA, Zurovac D, Snow RW (2009). Malaria drug shortages in Kenya: a major failure to provide access to effective treatment. Am J Trop Med Hyg.

[44] Bloland PB, Kachur SP, Williams HA (2003). Trends in antimalarial drug deployment in sub-Saharan Africa. J Exp Biol.

[45] Watsierah CA, Jura WGZO, Oyugi H, Abong’o B, Ouma C (2010). Factors determining anti-malarial drug use in a peri-urban population from malaria holoendemic region of western Kenya. Malar J.

